# The comparison of preoxygenation methods before endotracheal intubation: a network meta-analysis of randomized trials

**DOI:** 10.3389/fmed.2024.1379369

**Published:** 2024-06-07

**Authors:** Ming Zhong, Rong Xia, Junyu Zhou, Jing Zhang, Xia Yi, Anbo Yang

**Affiliations:** ^1^Department of Anesthesiology, The First Affiliated Hospital of Guangzhou University of Chinese Medicine, Guangzhou, China; ^2^Guangdong Clinical Research Academy of Chinese Medicine, Guangzhou, China; ^3^Chongqing Beibei Hospital of Traditional Chinese Medicine (Chongqing Hospital of The First Affiliated Hospital of Guangzhou University of Chinese Medicine), Chongqing, China

**Keywords:** preoxygenation, endotracheal intubation, network meta-analysis, ventilation, randomized clinical trial

## Abstract

**Background:**

Preoxygenation before endotracheal intubation (ETI) maintains asphyxiated oxygenation and reduces the risk of hypoxia-induced adverse events. Previous studies have compared various preoxygenation methods. However, network meta-analyses (NMAs) of the combined comparison of preoxygenation methods is still lacking.

**Methods:**

We searched for studies published in PubMed, Embase, Web of Science, Scopus, and the Cochrane Library. Review Manager version 5.3 was used to evaluate the risk of bias. The primary outcome of this meta-analysis was low oxygen saturation (SpO_2_) during ETI. The secondary outcomes included SpO_2_ <80%, SpO_2_ <90%, and apnea time during ETI. NMA was performed using R 4.1.2 software gemtc packages in RStudio.

**Results:**

A total of 15 randomized controlled trials were included in this study. Regarding the lowest SpO_2_, the noninvasive ventilation (NIV) with high-flow nasal cannula (HFNC) group performed better than the other groups. For SpO_2_ <80%, the NIV group (0.8603467) performed better than the HFNC (0.1373533) and conventional oxygen therapy (COT, 0.0023) groups, according to the surface under the cumulative ranking curve results. For SpO_2_ <90%, the NIV group (0.60932667) performed better than the HFNC (0.37888667) and COT (0.01178667) groups. With regard to apnea time, the HFNC group was superior to the COT group (mean difference: −50.05; 95% confidence interval: −90.01, −10.09; *P* = 0.01).

**Conclusion:**

Network analysis revealed that NIV for preoxygenation achieved higher SpO_2_ levels than HFNC and COT and offered a more significant advantage in maintaining patient oxygenation during ETI. Patients experienced a longer apnea time after HFNC preoxygenation. The combination of NIV with HFNC proved to be significantly superior to other methods. Given the scarcity of such studies, further research is needed to evaluate its effectiveness.

**Systematic review registration:**

identifier CRD42022346013

## Introduction

Invasive mechanical ventilation is a crucial measure to safeguard patient safety during surgical procedures conducted under general anesthesia, typically necessitating tracheal intubation. Prior to endotracheal intubation (ETI), the induction of anesthesia renders the patient unconscious, and neuromuscular blockade ensues, leading to hypopnea and apnea. This subsequent apnea period poses a heightened risk of hypoxia for the patient, particularly if tracheal intubation poses difficulties, further compounding existing risks (1, 2). Preoxygenation refers to the process of enhancing oxygen concentration and reserves by saturating the patient's body with oxygen prior to surgery, ensuring that the patient maintains a safe oxygen saturation (SpO_2_) level during apnea. Administering preoxygenation before ETI can sustain oxygenation during asphyxia and mitigate the hazards associated with hypoxia-induced adverse events. Consequently, it is highly advisable to routinely recommend preoxygenation as standard practice prior to ETI (3–5).

The most common form of preoxygenation is mask ventilation with 100% oxygen for 3–5 min, also known as conventional oxygen therapy (COT) (6). The simplicity of mask ventilation lies in its ease of operation; however, prolonged ventilation can compromise patient comfort. In addition to non-invasive ventilation (NIV), a novel approach, the high-flow nasal cannula (HFNC), has been increasingly employed in the preoxygenation process for patients in the operating room (7). HFNC administers heated and humidified gases through a nasal catheter, maintaining a specified fraction of inspired oxygen (FiO_2_) at a maximum flow rate exceeding 60 L/min. HFNC demonstrates satisfactory oxygenation effects, improves patient comfort, and ensures a more tolerable preoperative experience. Furthermore, mask ventilation obstructs the oral airway, necessitating the removal of the mask during laryngoscopy. This underscores the dual functionality of HFNC as a preoxygenation device capable of maintaining oxygenation during asphyxia. (8).

Previous studies have compared various preoxygenation methods, and some have suggested that HFNC is a more effective preoxygenation device (9, 10). In addition, according to the studies on NIV (11) and HFNC (12), preoxygenation is more effective than COT. Based on published studies, several meta-analyses have compared the effects of different preoxygenation modalities (13–15). A meta-analysis by Kuo et al. (15) reported that high-flow nasal oxygenation can enhance PaO_2_ and prolong safe apnea time. Li et al. (13) pointed out that transnasal humidified rapid-insufflation ventilatory exchange did not have a significant advantage over the use of facemasks, but it could effectively improve PaO_2_. According to Chiang et al. (14), NIV is more effective than conventional preoxygenation methods. Nonetheless, network meta-analyses (NMAs) comprehensively comparing different preoxygenation methods are scarce. Consequently, a systematic review of published studies along with an NMA assessing various preoxygenation modalities is necessary to provide a holistic understanding of their relative effectiveness and safety.

## Methods

### Study selection

**Figure 1:**
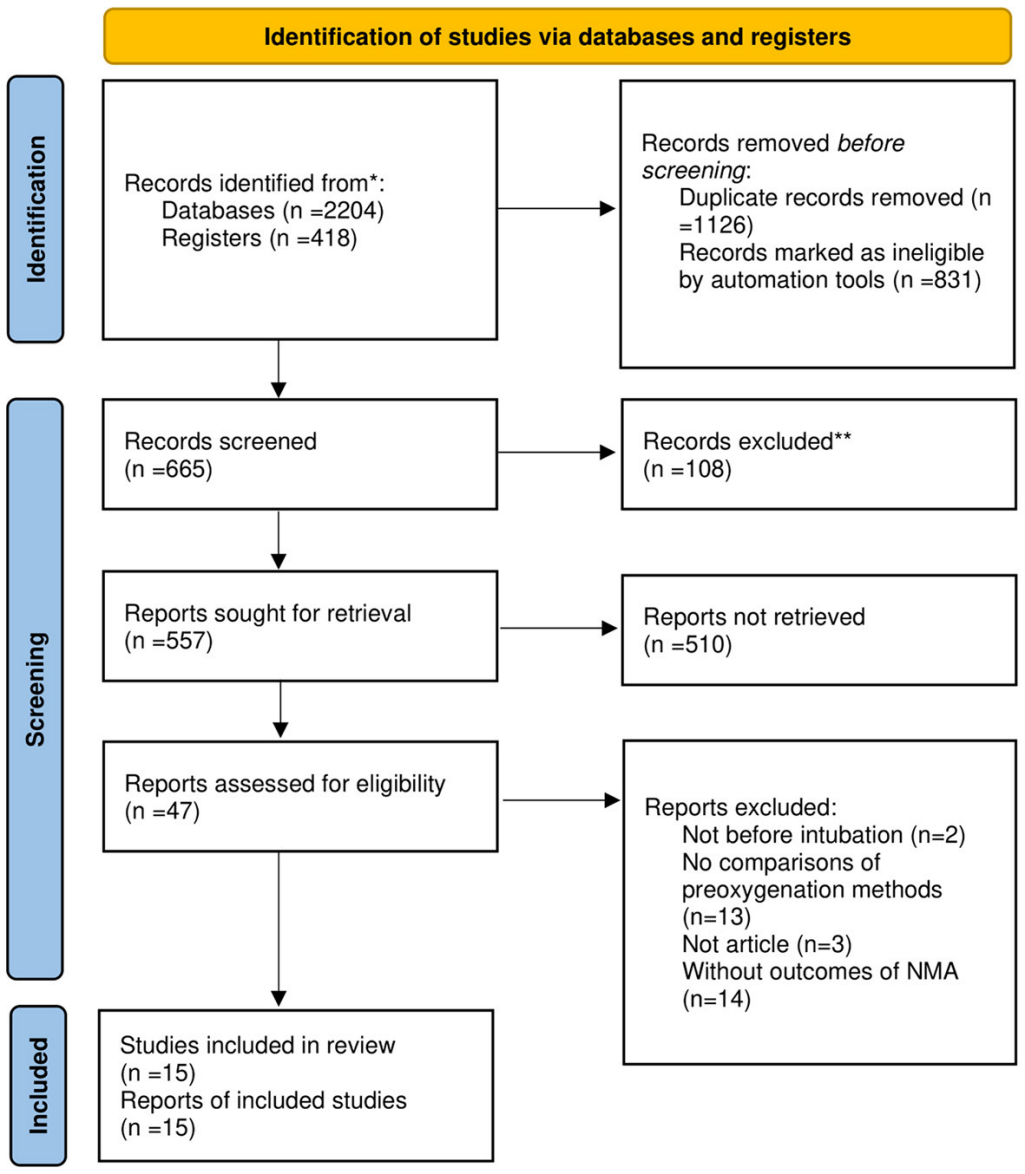
PRISMA flow diagram of the search strategy and included studies.

This systematic review and NMA has been registered with PROSPERO (registration number is CRD42022346013), and we performed it according to Preferred Reporting Items for Systematic Reviews and meta-analyses (PRISMA) guidelines. The researchers searched for studies from PubMed, Embase, Web of science, Scopus, and Cochrane library. The search terms are as follow: (“High-flow Nasal Cannula”[Title/Abstract] OR “HFNC”[Title/Abstract] OR “High flow nasal cannula therapy”[Title/Abstract] OR “nasal high flow”[Title/Abstract] OR “high flow nasal therapy”[Title/Abstract] OR “high flow oxygen therapy”[Title/Abstract] OR “high flow therapy”[Title/Abstract] OR “HFNO”[Title/Abstract] OR “high flow nasal oxygen”[Title/Abstract] OR “Non-invasive ventilation”[Title/Abstract] OR “NIV”[Title/Abstract] OR “Noninvasive Ventilation”[Title/Abstract] OR “helmet”[Title/Abstract] OR “face mask”[Title/Abstract] OR “Bag-valve mask”[Title/Abstract] OR “mask”[Title/Abstract] OR “conventional oxygen therapy”[Title/Abstract] OR “COT”[Title/Abstract] OR “facemask”[Title/Abstract] OR “nasal interface”[Title/Abstract] OR “bilevel positive airway pressure”[Title/Abstract] OR “BiPAP”[Title/Abstract] OR “continuous positive airway pressure”[Title/Abstract] OR “CPAP”[Title/Abstract] OR “low flow oxygen”[Title/Abstract] OR “standard nasal cannula”[Title/Abstract]) AND (“preoxygenation”[Title/Abstract] OR “apneic oxygenation”[Title/Abstract]). The searched literatures are managed with EndNote X9 (Thomson Reuters, NY, USA). Two investigators screened all studies, the flow diagram is shown in Figure 1. All disputes are resolved by AY.

### Eligibility criteria

We included RCTs involving adult patients who underwent preoxygenation prior to ETI. The preoxygenation devices included COT, NIV, and HFNC. Studies with the following characteristics were excluded: non-intubation; focus on only apneic oxygenation or ventilation; animal studies; protocols, reviews, guidelines, or conference abstracts; lack of control; and including healthy volunteers.

### Risk of bias assessment

**Figure 2:**
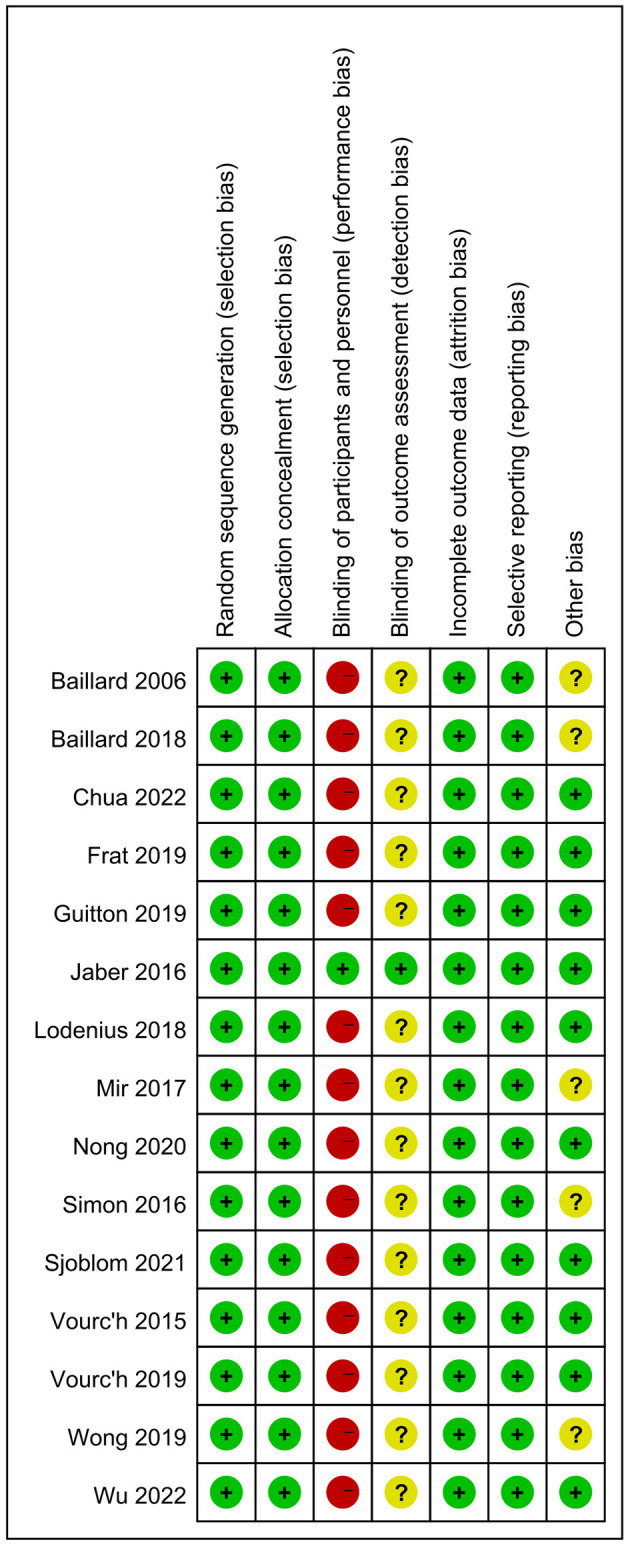
Risk of bias summary review authors' judgements about each risk of bias item for included RCTs.

Review Manager version 5.3 (RevMan 5.3) was used to evaluate the risk of bias in the included studies according to the Cochrane Collaboration tool. A summary of the risk of bias is shown in Figure 2. Three researchers (MZ, RX, and JZ) completed the risk of bias assessment, whereas the other researchers were responsible for deciding on a different opinion.

### Data extraction

Five investigators (MZ, RX, JZ, JZ, and XY) independently extracted data. MZ, RX, and JYZ. reviewed all the studies and excluded duplicates, registered studies, and nonclinical studies. Additionally, we reviewed the titles, abstracts, and full texts of the RCTs that were determined to be included in the NMA. XY summarized the characteristics of the 15 included studies in Table 1. AY were responsible for resolving disputes in the data extraction process.

**Table 1:** Characteristics of included RCTs.

**Study and published year**	**Participants**	**Preoxygenation in first group**	**Preoxygenation in second group**	**PaO_2_/FiO_2_ (mmHg) and PaO_2_ (mmHg) before intubation. Mean±SD or median [IQR]**
Baillard et al. (11)	*N =* 53 Inclusion criteria: Adults patients in ICUs with acute respiratory failure requiring intubation hypoxemia (PaO_2_ < 100 mm Hg with mask driven by 10 L/min oxygen). Exclusion criteria: Encephalopathy or coma, cardiac resucitation, and hyperkaliemia (> 5.5 mEq/L)	3-min preoxygenation with nonrebreather bag-valve mask driven by 15 L/min oxygen. Patients were allowed to breath spontaneously with occasional assistance.	3-min preoxygenation with NIV. PSV was delivered by an ICU ventilator through a face mask adjusted to obtain an expired tidal volume of 7 to 10 ml/kg. The FiO_2_ was 100% and PEEP level of 5 cm H_2_O.	PaO_2_: Control: 68 [60–79] NIV: 60 [57–89]
Baillard et al. (16)	*N =* 201 Inclusion criteria: Adults patients (age > 18) requiring intubation with hypoxemic acute respiratory failure. Exclusion criteria: Intubation for encephalopathy or coma, decompensation of chronic respiratory failure, cardiopulmonary resuscitation and pregnancy.	3-min preoxygenation with non-rebreathing bag-valve-mask with an oxygen reservoir driven by 15 L/min oxygen.	3-min preoxygenation with PSV was delivered by an ICU ventilator through a face mask adjusted to obtain an expired tidal volume of 6–8 ml/kg. The FiO_2_ was 100% and PEEP level of 5 cm H_2_O.	PaO_2_: Control: 126 [95–207] NIV: 132 [80–175]
Chua et al. (17)	*N =* 53 Inclusion criteria: Adults patients (age ≥ 21) requiring RSI due to any condition. Exclusion criteria: active “do-not-resuscitate” orders; crash, awake or delayed sequence intubations; requiring non-invasive positive pressure ventilation; cardiac arrest; suspicion or confirmed diagnosis of base of skull fractures or severe facial trauma that precluded placement of NC; pregnant women; and those incarcerated.	≥3 min of preoxygenation with usual care by preoxygenating using only non-rebreather mask at flush rate, and then given at least 15L/min of non-humidified and non-heated oxygen from wall supply via NC for apneic oxygenation.	≥3 min of preoxygenation with HFNC received 60L/min of warm and humidified oxygen at 37°C and FiO_2_ more than 0.90.	NR
Frat et al. (18)	*N =* 53 Inclusion criteria: Adults patients (age > 18) requiring intubation in the ICU with acute hypoxemic respiratory failure (RR > 25 bpm or signs of respiratory distress, PaO_2_/FiO_2_ < 300 mmHg regardless of oxygenation strategy). Exclusion criteria: Cardiac arrest, altered consciousness (GCS < 8).	3–5-min preoxygenation at 30°with HFNC with oxygen flow 60 L/min through a heated humidifier, FiO_2_ 1.0. Clinicians performed a jaw thrust to maintain a patent upper airway, and continued high-flow oxygen therapy during laryngoscopy until endotracheal tube was placed into the trachea	3–5-min preoxygenation at 30° with NIV-pressure support ventilation delivered via a face mask connected to an ICU ventilator, adjusted to obtain an expired tidal volume 6–8 ml/kg of predicted body weight with PEEP 5 cmH_2_O and FiO_2_ 1.0	PaO_2_/FiO_2_: HFNC: 148 ± 70 NIV: 142 ± 65
Guitton et al. (19)	*N =* 184 Inclusion criteria: Adults patients (age > 18) requiring intubation in the ICU, without severe hypoxemia (PaO_2_/FiO_2_ < 200 mmHg) Exclusion criteria: Intubation without RSI (cardiac arrest), fiberoptic intubation, asphyxia, nasopharyngeal blockade, grade 4 glottis on Cormack-Lehane scale	4-min preoxygenation in a head-up position with BVM (disposable self-inflating resuscitator with a reservoir bag, O_2_ set at 15 L/min)	4-min preoxygenation in a head-up position with HFNC (60 L/min flow of headed and humidified oxygen FiO_2_ 1.0, large or medium nasal cannula chosen according to patients' nostril size)	PaO_2_/ FiO_2_: BVM: 375 [276–446] HFNC: 318 [242–396]
Jaber et al. (20)	*N =* 49 Inclusion criteria: Patients with severe hypoxemic acute respiratory failure (RR > 30 bpm, FiO_2_ requirement ≥ 50% to obtain > 90% SpO_2_ or an impossibility to obtain > 90% SpO_2_, estimated PaO_2_/FIO_2_ < 300 mmHg) admitted to ICU requiring mechanical ventilation. Exclusion criteria: Cardiocirculatory arrest.	4-min 30° head-up inclination with HFNC (humidified O_2_ flow 60 L/min, FiO_2_ 100%) combined with NIV (PS 10 cmH_2_O, PEEP 5 cmH_2_O, FiO_2_ 100%)	4 min 30° head-up inclination with NIV (PS 10 cmH_2_O, PEEP 5 cmH_2_O, FiO_2_ 100%)	PaO_2_/ FiO_2_: HFNC with NIV: 107 [74–264] NIV: 140 [83–201]
Lodenius et al. (21)	*N =* 79 Inclusion criteria: Adult patients (> 18 years) who required RSI of anesthesia for emergency surgery during daytime hours. Exclusion criteria: BMI > 35 kg.m^−2^, pregnancy, a need for non-invasive ventilation to maintain oxygenation, or inability to give consent.	≥3 min of preoxygenation with a tightly held facemask and a fresh gas flow of 10 L/min delivered via a circle system (FiO_2_ 100%).	≥3 min of preoxygenation with a flow of 40 L/min with heated and humidified 100% oxygen via a nasal cannula was delivered.	NR
Mir et al. (9)	*N =* 40 Inclusion criteria: Patients who required rapid sequence induction of general anesthesia for emergency surgery, whose routine clinical care required arterial blood gas sampling, and who were competent to give consent were recruited. Exclusion criteria: patients < 16 years, unable to give informed consent because of a language barrier, or had severe respiratory disease.	3 min of preoxygenation with HFNC. The oxygen flow rate was started at 30 L/min, and was increased to 70 L/min over the course of the first minute of pre-oxygenation.	3 min of preoxygenation with facemask using a circle system with an oxygen rate of 12 L/min.	NR
Nong et al. (22)	*N =* 106 Inclusion criteria: Adults patients (age > 18) requiring intubation in the ICU. Attending physician based on worsening respiratory failure (e.g., blood oxygen saturation (SpO_2_) < 88% and RR > 36/min) after adequate therapy, fraction of inspired oxygen (FiO_2_) > 60%, intolerance to NIV, neurological deterioration, or copious respiratory secretions. Exclusion criteria: age < 18 years, pregnancy, severe coagulopathy, cardiac arrest, and contraindications for bag-valve-mask or NIV preoxygenation.	≥3 min of preoxygenation with bag-valve-mask driven by 15 L/min oxygen flow, an oxygen reservoir was added to the balloon, and positive end-expiratory pressure was set at 5 cmH_2_O.	≥3 min of preoxygenation with NIV support with the following settings: mode, S/T; f, 20/min; inspiratory positive airway pressure, 12–20 cmH_2_O (adjusted to obtain an expired tidal volume of 7–10 ml/kg); expiratory positive airway pressure, 5 cmH_2_O; and FiO_2_, 100%.	PaO_2_: Control: 75.1 ± 41.0 NIV: 75.8 ± 32.6
Simon et al. (12)	*N =* 40 Inclusion criteria: Adults patients (age≥ 18) with respiratory failure with hypoxemia (PaO_2_/FiO_2_ < 300 mmHg), indicated for endotracheal intubation. Exclusion criteria: Difficult airway, nasopharyngeal obstruction or blockage	3-min preoxygenation using a BVM (adult size AMBU SPUR II disposable resuscitator with oxygen bag reservoir and without PEEP valve or pressure manometer), O_2_ 10 L/min. No manual insufflation performed during apneic period.	3-min preoxygenation using HFNC, oxygen flow 50 L/min, FiO_2_ 1.0; left in place during the intubation procedure.	PaO_2_/ FiO_2_: BVM: 205 ± 59 HFNC: 200 ± 57
Sjöblom et al. (10)	*N =* 349 Inclusion criteria: Adult patients requiring intubation for emergency surgery. Exclusion criteria: BMI > 35 kg.m^−2^; pregnancy; need for non-invasive ventilation before anesthesia; or not reaching SpO_2_ > 93% during pre-oxygenation.	≥3 min of preoxygenation with a tight-fitting facemask with a fresh gas flow of 10 L/min delivered via a circle system.	≥3 minu of preoxygenation with HFNC in 30–50 L/min of heated and humidified oxygen.	NR
Vourc'h et al. (23)	*N =* 119 Inclusion criteria: Adults (≥18 years) with acute hypoxemic respiratory failure (RR> 30 bpm and FiO_2_ ≥ 50% to obtain > 90% oxygen saturation, and estimated PaO_2_/FiO_2_ < 300 mmHg) requiring endotracheal intubation in ICU after RSI. Exclusion criteria: Cardiac arrest, asphyxia, intubation without RSI, Cormack-Lehane grade 4 glottis	4 min of preoxygenation with high FiO_2_ facial mask (15 L/min oxygen flow).	4 min of preoxygenation with HFNC set to 60 L/min of humidified oxygen flow (FiO_2_ 100 %).	PaO_2_/ FiO_2_: Facial mask: 115.7 ± 63 HFNC: 120.2 ± 55.7
Vourc'h et al. (24)	*N =* 100 Inclusion criteria: Adults with a BMI> 35 kg/m^2^ and a planned RSI airway control strategy. Exclusion criteria: Age < 18 years, SpO_2_ <90% in air, haemodynamic instability, patients admitted for burns, intubation without laryngoscopy (i.e., fibreoptic intubation for anticipated “cannot ventilate situation” or mouth opening < 2 cm), Grade 4 glottis exposure on the Cormack-Lehane scale documented during a previous anesthesia, adults subject to legal protection, pregnancy, lack of consent, patients without French health insurance, or already participating in an interventional study on preoxygenation.	4 min of preoxygenation with Face Mask connected to an Aisys CS2 ventilation system. In this group, ventilator was set on pressure support mode with expiratory positive airway pressure (EPAP) of 5 cm H_2_O and inspiratory positive airway pressure (IPAP) of 15 cmH_2_O, meaning a 10 cm H_2_O pressure support, FiO_2_ 100%	4 min of preoxygenation with HFNC, nasal prongs set at 60 L/min flow of heated and humidified pure oxygen (FiO_2_ 100%, 37 °C).	PaO_2_: NIV: 99 [97–100] HFNC: 97 [97–99]
Wong et al. (25)	*N =* 40 Inclusion criteria: Adult patients 18 years or older with body mass index ≥40 kg·m^−2^ scheduled for elective surgery under general anesthesia requiring tracheal intubation. Exclusion criteria: Included moderate to severe comorbidity (severe chronic respiratory or renal disease, uncontrolled hypertension or ischemic heart disease, increased intracranial pressure), uncontrolled gastric reflux disease, anticipated or history of difficult airway or inability to breathe through nose due to nasopharyngeal obstruction and compulsive mouth breather.	The control group patients received preoxygenation with FIO_2_ of 1.0 using a facemask at 15 L/minute until the end-tidal oxygen was >85% for 3 min.	The high-flow nasal oxygenation group patients were preoxygenated with high-flow nasal cannula at 40 L/minute of FIO_2_ 1.0 for 3 min	NR
Wu et al. (26)	*N =* 80 Inclusion criteria: age 20 to 65 years, and BMI equal to or higher than 30 kg·m^−2^. Exclusion criteria: SpO_2_ <90% in room air, severe cardiopulmonary disease (e.g., left ventricular ejection fraction < 40%, diagnosed coronary artery disease, and aortic dissection), hemodynamic instability, renal insufficiency (estimated glomerular filtration rate < 30 mL·min·1.73 m^−2^), pregnancy, and patient refusal.	Preoxygenation was performed using a size−3 or −4 fitting anesthetic facemask, connected to a ventilation system with 100% oxygen 15 L/min.	Preoxygenation was performed using a HFNC, with nasal prongs set at 30 L/min flow of heated and humidified 100% oxygen.	PaO_2_: Facemask: 84 [76–93] HFNC: 90 [81–97]

FiO_2_, fraction of inspired oxygen; PSV, pressure support ventilation; RSI, rapid sequence intubation; HFNC, high-flow nasal cannula; NIV, non-invasive ventilation; NC, nasal cannula; NR, no record; RR, respiratory rate; PS, pressure support; BMI, body mass index.

### Outcomes

The primary outcome of this meta-analysis was low SpO_2_ during ETI. The secondary outcomes included SpO_2_ <80%, SpO_2_ <90%, and apnea time during ETI. We reported the odds ratios (ORs), mean differences (MDs), and 95% confidence intervals (CI) in a pairwise meta-analysis. The log-OR, MD, and 95% CI were reported for the NMA.

### Statistical analysis

Five investigators (MZ, RX, JZ, JZ, and XY) used statistical methodology. First, RevMan 5.3 was used for pairwise meta-analysis. For the heterogeneity test, when *P* < 0.05 or I2 > 50%, we chose the random-effects model. When the heterogeneity test yielded *P* > 0.05 or I2 < 50%, the fixed effects model was often selected.

Second, STATA (version 17.0) was used to generate network plots for the different groups, to visualize the relationships between various interventions. The size of the node in the network plot represents the sample size of the group, and the edge width represents the number of studies.

Third, NMA was performed using R 4.1.2 software gemtc packages in RStudio, based on a Bayesian framework with Markov Chain Monte Carlo (MCMC) simulation. We ran the estimation with a burn-in of 25,000 iterations and sampling of 50,000 iterations from the three chains of initial values. The selection between models was based on the Deviance Information Criteria (DIC). A DIC difference in the consistency test results >5 was considered significant. The fluctuation process of the MCMC chain is represented by a trace plot and the convergence degree of the model is diagnosed together with the density and Brooks-Gelman-Rubin diagnosis plots with the potential scale reduction factor (PSRF). If the degree of model convergence is poor or 1 < PSRF ≤ 1.05, the frequency of pre-iteration and iterations need to be changed.

Fourth, we used R 4.1.2 software to calculate the surface under the cumulative ranking (SUCRA) to rank the interventions. A heatmap was used to visualize the SUCRA results. Results of two-tailed tests with P < 0.05 were considered statistically significant.

## Results

### Literature search

We searched five databases for 2,622 studies (Databases: 2,204; Registers: 418). After a review by two investigators, 15 RCTs were included in the systematic review and NMA. The search details are represented in a flow diagram in Figure 1.

### Study characteristics

The characteristics of the 15 included RCTs, including the study and publication year, participants, and intervention characteristics, are summarized in Table 1. All the studies were published after 2000 and the sample size for each group was at least 20. In addition, the preoxygenation maintenance time was 3–5 min.

### Risk of bias assessment and study confidence rating

The risk of bias assessment is shown in Figure 2. All the studies reported random sequence generation and allocation concealment. Blinding was defined as impossible in the included studies; therefore, performance bias for 14 RCTs was defined as high risk, and outcome assessments were defined as unclear owing to a lack of reporting. However, blinding was achievable in a study by Jaber et al. (20); hence, the risk of bias for blinding was defined as low.

### Network plot of eligible comparisons of outcomes

**Figure 3:**
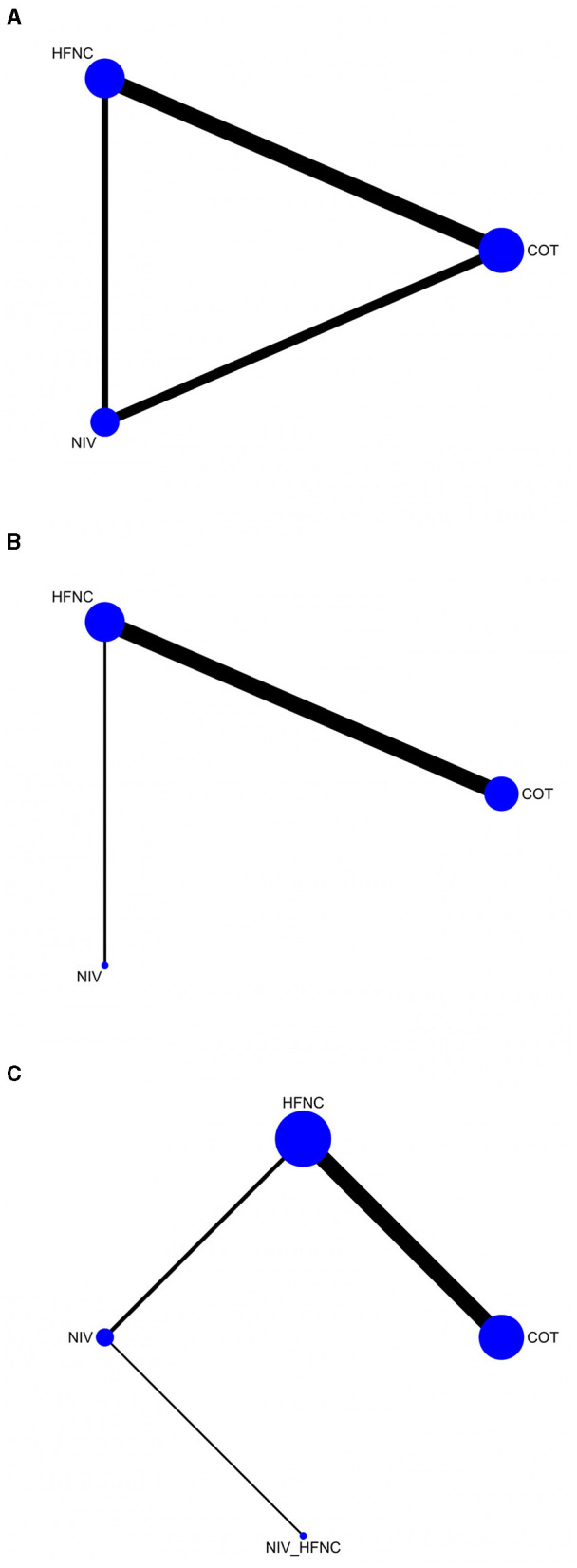
The network geometry of SpO_2_ <80% during ETI procedure **(A)**; SpO_2_ <90% during ETI procedure **(B)**; Lowest SpO_2_ during ETI procedure **(C)**.

Network maps of the outcomes are shown in Figure 3. The outcomes include SpO_2_ <80% during ETI (Figure 3A), SpO_2_ <90% during ETI (Figure 3B), and the lowest SpO_2_ during ETI (Figure 3C).

### SpO_2_ <80% during ETI procedure

A total of 10 RCTs (10–12, 16, 18, 19, 21–24) (1,544 patients) reported SpO_2_ <80% during the ETI procedure. According to the pairwise meta-analysis results shown in Table 2, the NIV group was superior to the COT group (OR: 4.46; 95% CI: 1.29; 15.43, *P* = 0.02).

**Table 2:** The direct evidence from pairwise meta-analysis and heterogeneity of outcomes.

**Comparison**	**Number study**	**OR**	**95% CI**	***P*-value**	**I^2^ statistic**
SpO_2_ <80% during ETI procedure				
COT vs. NIV	3	4.46	1.29, 15.43	0.02	73%
COT vs. HFNC	5	1.36	0.74, 2.49	0.32	0%
NIV vs. HFNC	2	0.80	0.49, 1.33	0.40	0%
SpO_2_ <90% during ETI procedure				
COT vs. HFNC	6	1.87	1.23, 2.82	0.003	4%
NIV vs. HFNC	1	0.78	0.02, 3.10	0.73	NE
Lowest SpO_2_ during ETI procedure				
COT vs. HFNC	8	−0.77	−1.49, −0.05	0.04	56%
NIV vs. HFNC	2	1.31	0.05, 2.57	0.04	20%
NIV vs. NIV_HFNC	1	−4.00	−6.53, −1.47	0.002	NE
Apnoea time (s)					
COT vs. HFNC	4	−50.05	−90.01, −10.09	0.01	92%

OR, Odd Ratio; CI, Confidence Interval; NE, Not Estimate; NIV, Noninvasive Ventilation; COT, conventional oxygen therapy; HFNC, High Flow Nasal Cannula; ETI, Endotracheal Intubation.

**Figure 4:**
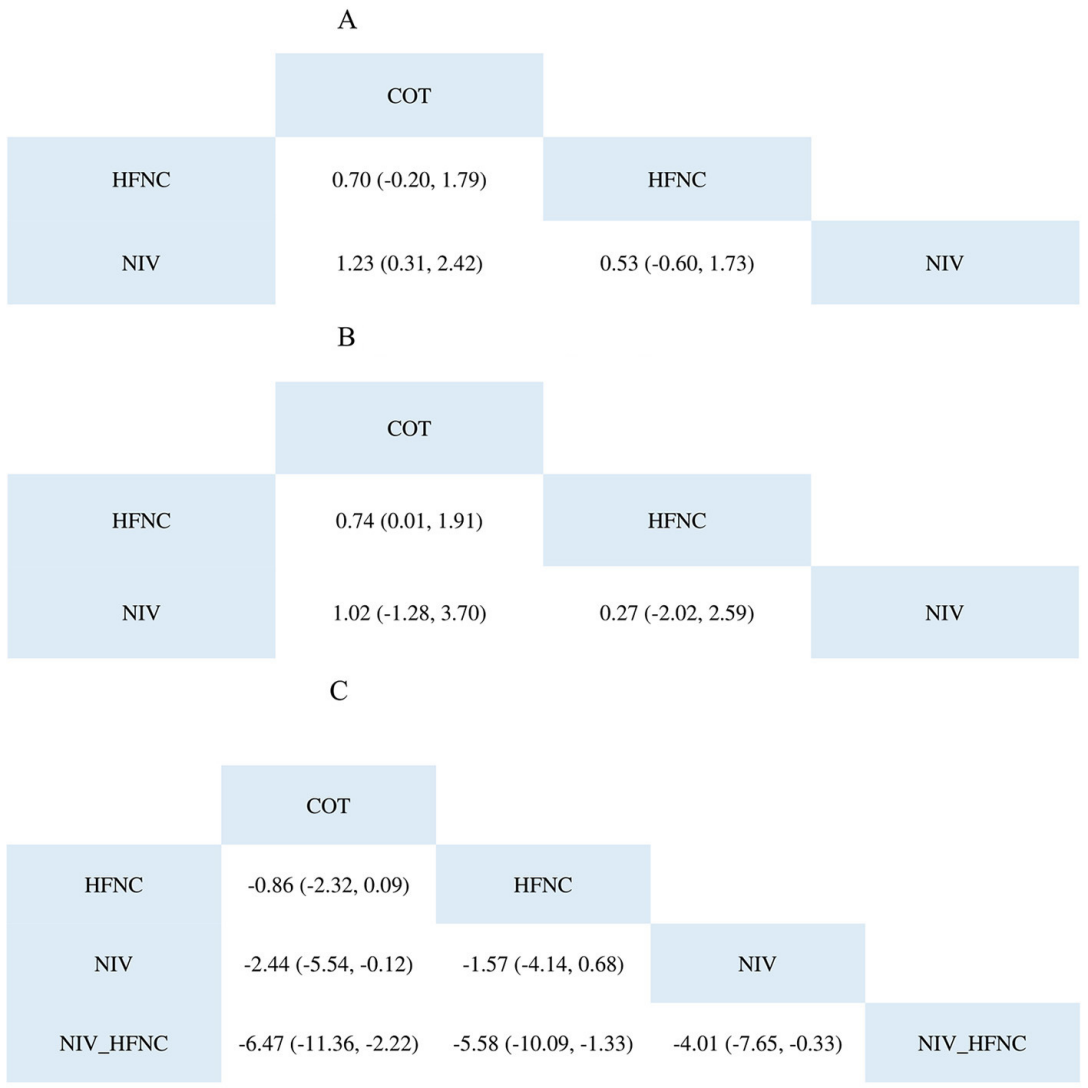
The league table of SpO_2_ <80% during ETI procedure **(A)**; SpO_2_ <90% during ETI procedure **(B)**; Lowest SpO_2_ during ETI procedure **(C)**.

The NMA results (Figure 4A) showed that the NIV group was superior to the COT group (Log OR 1.23; 95% CI: 0.31, 2.42). For the SUCRA results shown in Table 3, the NIV group (0.8603467) performed better than the HFNC (0.1373533) and COT (0.0023) groups.

**Table 3:** The SUCRA results of network analysis outcomes.

**Intervention**	**SpO_2_ <80% during ETI procedure**	**SpO_2_ <90% during ETI procedure**	**Lowest SpO_2_ during ETI procedure**
HFNC	0.1373533	0.37888667	0.004345
NIV	0.8603467	0.60932667	0.01541
COT	0.0023	0.01178667	0.000545
NIV_HFNC	NA	NA	0.9797

NIV, Noninvasive Ventilation; COT, conventional oxygen therapy; HFNC, High Flow Nasal Cannula; ETI, Endotracheal Intubation; SUCRA, surface under the cumulative ranking curve.

### SpO_2_ <90% during ETI procedure

Seven RCTs (10, 17, 19, 21, 23, 24, 26) (1,101 patients) reported SpO_2_ <90% during the ETI procedure. According to the pairwise meta-analysis results shown in Table 2, the HFNC group was superior to the COT group (OR: 1.87; 95% CI: 1.23, 2.82; *P* = 0.003).

The NMA results (Figure 4B) showed that the HFNC group was superior to the COT group (Log OR 0.74; 95% CI: 0.01, 1.91). Regarding the SUCRA results, as shown in Table 3, the NIV group (0.60932667) performed better than the HFNC (0.37888667) and COT (0.01178667) groups.

### Lowest SpO_2_ during ETI procedure

Eleven RCTs (10, 12, 17–21, 23–26) (1,543 patients) reported the lowest SpO_2_ during the ETI procedure. Table 2 presents the results of the pairwise meta-analysis. The HFNC group was superior to the COT group (MD: −0.77; 95% CI: −1.49, −0.05; *P* = 0.04), the NIV group was superior to the HFNC group (MD: 1.31; 95% CI: 0.05, 2.57; *P* = 0.04), and the NIV with HFNC group was better than the NIV group (MD: −4.00; 95% CI: −6.53, −1.47; *P* = 0.002).

According to the NMA results (Figure 4C), the NIV group was superior to the COT group (MD: −2.44; 95% CI: −5.54, −0.12), whereas the NIV with HFNC group was better than the other groups. The SUCRA results are presented in a heatmap (Table 3). The NIV with HFNC group (0.9797) was better than the NIV (0.01541), HFNC (0.004345), and COT (0.000545) groups.

### Apnoea time

Four RCTs (9, 10, 21, 25) (508 patients) reported apnea time during the ETI procedure. According to the pairwise meta-analysis results, the HFNC group had a longer apnea time (MD: −50.05; 95% CI: −90.01, −10.09; *P* = 0.01) than the other groups.

## Discussion

This study marks the first comparison of multiple preoxygenation techniques using NMA. The findings revealed that NIV coupled with HFNC effectively maintained high SpO_2_ levels during ETI. Both NIV and HFNC significantly reduced the risk of SpO_2_ dipping to <80% and <90%, respectively, with NIV demonstrating superiority over both HFNC and COT. Furthermore, the use of HFNC for preoxygenation extended the duration of apnea.

Preoxygenation is a crucial measure for safeguarding patient oxygenation throughout the intubation process and ensuring surgical safety, particularly in individuals anticipated to encounter airway challenges. Both the 2015 Difficult Airway Society guidelines and 2022 American Society of Anesthesiologists recommendations emphasize the significance of this practice (6, 27). Among them, the 2022 guidelines recommend 3 min of preoxygenation to reach an end-tidal oxygen concentration of 0.90 or higher (EtO_2_ ≥ 0.9) and use of various NIV devices, such as nasal catheters and masks. As a modification of conventional nasal catheters, HFNC can deliver high-flow oxygen, generate low levels of positive end-expiratory pressure (PEEP), and allow asphyxiation and oxygenation, making it useful for preoxygenation or oxygen therapy (28). Despite the obvious benefits of HFNC in patients with acute hypoxic respiratory failure and after scheduled extubation (10, 23), its effectiveness is still controversial compared with other preoxygenation methods (29, 30). Guitton et al. (19) reported that HFNC preoxygenation reduced tracheal intubation-related adverse events but did not improve lowest SpO_2_. In addition, Vourc'h et al. (23) compared preoxygenation methods in obese patients and found that the HFNC group had a significant advantage in the lowest SpO_2_ compared with the NIV group. We included the study by Jaber et al. (20) on NIV combined with HFNC for preintubation oxygenation in critically ill patients with severe hypoxemia and acute respiratory failure. The results of our study indicate that when used in combination with preoxygenation, NIV and HFNC were associated with the lowest SpO_2_ levels. Paradoxically, our findings suggest that the combined use of NIV and HFNC could potentially improve the lowest SpO_2_ recorded. However, because of the scarcity of relevant studies, our analysis was limited to only one study, thus precluding a direct comparison. Consequently, further studies are required to comprehensively evaluate the effects of this combined approach on preoxygenation. In addition, owing to the small sample size, treatment effects and publication bias need to be considered; therefore, caution must be exercised when interpreting the results of HFNC and NIV studies. Additionally, the best method for combining HFNC and NIV to reduce air leakage has not been well described. Whether continuous nasal positive airway pressure masks play a special role in preoxygenation is worth exploring.

When comparing SpO_2_ <90% and SpO_2_ <80%, we found no significant advantage of HFNC over NIV, although both were superior to COT. A multicenter, randomized, open-label trial reported the effect of preoxygenation in patients with acute respiratory failure, showing that neither NIV nor HFNC changed severe hypoxemia in these patients, with no significant difference between them (18). A study designed by Kuo et al. pointed out that HFNC can better enhance PaO_2_ and has an advantage over mask oxygenation in preventing ETI (15). The reasons for this difference in outcomes may be patient characteristics and oxygen flow settings. Some studies have pointed out that NIV may be a better method of preoxygenation for obese patients (31, 32), and HFNC preoxygenation patients have lower EtO_2_ and SpO_2_ levels than the NIV group. This result may be related to the limited supraglottic pressure produced by HFNC, the inability to repair airway obstruction after general anesthesia, and the difficulty in maintaining or restoring FRC damage in obese patients (33). However, considering that HFNC is better tolerated and has a median SpO_2_, it is an acceptable alternative for obese patients without NIV or with contraindications (24, 34). In addition, several studies have reported the comparison of preoxygenation methods in critically ill patients with hypoxemia or acute respiratory failure (11, 12, 16, 23). In 2019, Fong et al. (35) conducted an NMA of RCTs on the effects of preoxygenation in patients with acute hypoxic respiratory failure. Seven RCTs encompassing 959 patients were comprehensively analyzed in this study. These findings indicate that NIV is a safe and potentially the most effective preoxygenation technique. One explanation for the inferior performance of HFNC could be the loss of the PEEP effect in patients experiencing respiratory distress due to mouth opening (36). In these patients, the nasal and oral inhalation flows can be as high as 110 and 280 L/min, respectively, which are significantly better than those of HFNC (37). Another possible explanation is that NIV allows the delivery of high levels of FiO_2_ and positive intrathoracic pressure, promoting alveolar replenishment, which may improve the efficiency of gas exchange (38, 39).

This study has some limitations. Constrained by the paucity of RCTs reporting pertinent comparisons, only 15 studies were included in this analysis, potentially introducing a reporting bias. Furthermore, the combined use of NIV and HFNC for preoxygenation was reported in a single study. Although the initial results appear promising, the absence of a direct comparative evidence necessitates cautious interpretation of the conclusions.

## Conclusions

The results of the network analysis showed that NIV for preoxygenation achieved a higher SpO_2_ than HFNC and COT and had a more significant benefit in maintaining patient oxygenation during ETI. Patients had a longer apnea time after HFNC preoxygenation. The effect of NIV combined with HFNC was significantly better than that of other methods. Owing to a lack of studies, further investigation is warranted to evaluate their effects in the future.

## Data availability statement

The original contributions presented in the study are included in the article/supplementary material, further inquiries can be directed to the corresponding author.

## Author contributions

MZ: Conceptualization, Data curation, Formal analysis, Investigation, Methodology, Project administration, Software, Supervision, Validation, Visualization, Writing—original draft, Writing—review & editing. RX: Conceptualization, Data curation, Formal analysis, Investigation, Methodology, Project administration, Software, Writing—original draft, Writing—review & editing. JZho: Conceptualization, Data curation, Formal analysis, Investigation, Methodology, Writing—original draft, Writing—review & editing. JZha: Conceptualization, Data curation, Formal analysis, Methodology, Software, Writing—original draft, Writing—review & editing. XY: Conceptualization, Formal analysis, Investigation, Methodology, Writing—original draft, Writing—review & editing. AY: Project administration, Supervision, Validation, Writing—review & editing.
